# Constructivist developmental theory is needed in developmental neuroscience

**DOI:** 10.1038/npjscilearn.2016.16

**Published:** 2016-12-14

**Authors:** Marie Arsalidou, Juan Pascual-Leone

**Affiliations:** 1 Department of Psychology, National Research University Higher School of Economics, Moscow, Russia; 2 Department of Psychology, York University, Toronto, ON, Canada

## Abstract

Neuroscience techniques provide an open window previously unavailable to the origin of thoughts and actions in children. Developmental cognitive neuroscience is booming, and knowledge from human brain mapping is finding its way into education and pediatric practice. Promises of application in developmental cognitive neuroscience rests however on better theory-guided data interpretation. Massive amounts of neuroimaging data from children are being processed, yet published studies often do not frame their work within developmental models—in detriment, we believe, to progress in this field. Here we describe some core challenges in interpreting the data from developmental cognitive neuroscience, and advocate the use of constructivist developmental theories of human cognition with a neuroscience interpretation.

## Introduction

Historically, the study of cognition has focused mainly on adults. With the arrival of constructivist theoreticians such as Jean Piaget, cognitive developmental theory became more visible in psychology. Piaget observed that children’s cognitive development undergoes several stages marked by developmental milestones attained by children at each of these levels. Such theoretical and practically important stages are indexing series of functional metamorphoses (i.e., constructivist-developmental processes) that finally change children into grown adults. Merely testing children is not enough to understand functional stages of development; theories are fundamental because developmental stages are not equivalent to developmental states. Concepts of states and stages are confused in the literature, and this might explain some researchers’ misunderstanding. As used by Piaget and other developmental constructivists, a state is a pure description of a here-and-now manifest performance or internal processing of the organism. In contrast a stage is a sequence of descriptive states (descriptive models), each coupled with its corresponding (specific) causal-process model. Theory-based developmental research is critical for advancing both developmental psychology and developmental neuroscience. In the present manuscript we will focus on benefits of developmental constructivist theory for developmental neuroscience.

Piaget developed his theory, almost 100 years ago, without the benefit of current neuroscience. His constructivist models pioneered a differential experimental–developmental analysis of stages. Non-invasive neuroscience techniques such as magnetic resonance imaging (MRI), functional MRI, electroencephalography (EEG) and event-related potentials (ERP), provide the data on children’s neural processing within tasks—a great advance, severely limited by the scarcity of clear (and developmentally anchored in behaviour) theoretical models of how brain processes underlie human performances. Critically, at present, many observed developmental differences are interpreted *post hoc*, based on reverse inference on how they compare with adults’ activation.^1^ A developmental perspective guided by theoretical models might facilitate interpretation of brain processes related to tasks, which in turn could serve to revise, and explicate analytically ‘from within’, developmental process models. These models distinguish between information-bearing schemes (circuits and networks) and general-purpose (non-informational) regulations or capacities of the brain—which we call hidden operators, such as mental/executive attention or working memory, or associative versus constructivist learning and so on. Representations and performances, in developmental constructivist models, are overdetermined by the currently activated compatible schemes—aided by general-purpose brain capacities.^2,3^ Constructivist-developmental processes and their stages (as distinct from, but complementary with, learning and neuroplasticity) are heuristically important to understand relations between child behaviour and brain development. They should be a part of neuroscience research designs. We present examples of current developmental cognitive neuroscience and explain why neuroscience research will be less productive if children are studied without a grounded developmental method and developmental theories.

## General state of developmental cognitive neuroscience

Neuroimaging allows mappings of brain processes to cognitive performance. Nonetheless, even with healthy and typically developing children, authors often reach inconsistent conclusions. For instance, when tested with cognitive tasks evidence shows that similar regions become active for children and adults.^4^ However, in some studies researchers find a wider set of areas activated for children than in adults,^5^ but in other studies the number of brain regions showing linear load dependency expands with increasing age.^6^ Studies also show that children who exhibit better performance on cognitive tasks exhibit more activity in task-relevant brain regions,^7^ although better performance is associated as well with fewer activated regions.^8^ These results would benefit from a developmental framework that explains the global and less differentiated performance in younger children in terms of processing limits in mental/executive attentional capacity in younger children.^2,9–14^ Overall, brain responses are affected by chronological age and performance scores, although the relation often is not semantically specified.

Inconsistent findings are also revealed when examining relations between cortical and functional indices of development. For instance, thinner cortices in children and adolescents relate to either increased or decreased activation, depending on brain location and performance improvement.^15^ Similarly, thinner right inferior frontal cortices are linked to increased right inferior frontal activation in children and adolescents.^16^ However, in a larger sample of adolescents no relation is observed between cortical and functional maturation—with authors cautioning against pre-assuming such an association.^17^ To make matters more interesting, hemispheric involvement is not easily predictable in children; for instance, in response to cognitive tasks children and adolescents show less refined and less lateralised patterns than adults in one study^18^ and similar lateralisation patterns to adults in another.^19^ Another study revealed cognitive load to influence laterality as a function of age, but only for the right hemisphere.^20^ Heterogeneous findings in hemispheric asymmetry would benefit from a constructivist developmental interpretation that explains hemisphere involvement in terms of the task’s mental-demand (i.e., processes and difficulty) and mental-attentional capacity of the individual,^14,21,22^ congruent with evidence from developmental change in coherence measures in electroencephalography.^23,24^ Specifically, tasks that are too easy or too difficult tend to favour the right hemisphere, whereas problem-solving (often misleading) tasks that are within individuals’ limit in mental-attentional capacity favour the left hemisphere. During development, both mental-attentional capacity limits and learned automatisations grow with age; thus lateralisation elicited by tasks should be relative to *both* age and task characteristics. Further, as Nunez *et al.*^16^ found ‘children with thicker cortex in the right IFG (inferior frontal gyrus) rely less on right hemisphere language areas to process linguistic/syntactic information, relying more on left hemisphere processing than children with thinner cortex in the same brain regions.’ Thicker grey matter in typical children’s cortex is likely to express immaturity, whereas thinner areas may indicate that the learning-contingent maturational pruning has taken place^25^—i.e., local processing has matured. Note that adult theories cannot make age-contingent predictions because they lack constructivist developmental mechanisms.^22,26,27^

Neuroscience research with children and adolescents also examines how cognition relates to resting state, biogenetics and learning. Chai *et al.*^28^ examined data from participants aged 8–24 years showing a change in relative rate of active functional connectivity between medial and dorsolateral prefrontal cortices; cortices that relate, respectively, to default mode and executive task-relevant networks. This default-net-to-executive-net correlation actually reverses from being positive in children to negative in adults. Such interesting finding would benefit from a constructivist developmental explanation. Specifically, the younger are the children the more their behaviour should tend to be directly driven by affects, feelings and emotions (executive processes being often tied to these personal determinants); which should shift activity to their medial areas of the brain. In contrast, executive processes in older children and adults have already become main determinants of ordinary behaviour; which allows them ordinarily to control/inhibit personal processes^29^—producing the negative correlation found in adults.

Prefrontal functioning associated with the attentional network is also influenced by other genetic factors. Children with biogenetic variants within the dopamine D4 receptor gene (DRD4), with the seven-repeat allele present or absent, were tested with fMRI and cognitive tasks.^30^ Although no behavioural differences were observed across groups, children with the seven-repeat allele had lower neural activation than children without the seven-repeat allele in the left middle and inferior frontal gyri, showing an association between genotype and prefrontal functioning.^30^ A constructivist developmental theory may explain these finding in terms of developmental growth of mental/executive attention (both activatory and inhibitory capacity), a growth that may be related to the Dopamine D4—inhibitory—receptor gene.^31–33^ If the seven-repeat allele potentiates this mental/executive attentional inhibition and so adds potency to each quantum of mental attention, then the left frontal gyrus could remain less activated in children with seven-repeat allele for the same cognitive performance gain.^34^ Indeed, although they interpret the findings somewhat differently, Peterson and Posner^32^ highlight the developmental significance of Dopamine D4 receptor gene when they write: ‘One study found that only those children with the 7-repeat of the DRD4 showed the influence of a parent training intervention study’.^35^

Learning is an intrinsic part of development, albeit distinct from maturation of mental attentional capacity.^2,14,34,36^ A study of participants aged 6 to 20 years shows that (compared with cognitive measures closely tied to past learning) the indices of structural maturation and brain activity are better able to predict cognitive performance 2 years later.^37^ It is interesting to note that regions predictive of future cognitive performance are associated with structures such as the basal ganglia and thalamus (prewired twin coordinators of action/operative processes versus representational/figurative processes, respectively), rather than the typical attentional-network areas such as prefrontal and parietal cortices.^37^ A similar conclusion is drawn from a study examining mathematical achievement, which shows that individual responses to 8 weeks of training can be predicted by brain anatomy and functional connectivity measures recorded before training; this is the connectivity and cortical indices of hippocampus and basal ganglia, but not of parietal areas—sites of learning typically engaged during mathematical problem solving.^38^ These cognitive neuroscience findings can inform, and be informed by, developmental theorising; they support theories that explain learning in terms of brain-operator processes that are not content- or material-specific (e.g., visual-spatial or verbal), because these brain regions (basal ganglia, thalamus and hippocampus) have complex coordinating functions that apply across content domains. Problem solving and complex learning processes are clearly mediated in the brain through prefrontal-basal–ganglia–thalamus pathways.^39^ The subcortical (maturational) substratum of mental attention takes therefore precedence for predicting children’s cognitive learning.

Overall, although often in conflict, findings of developmental cognitive neuroscience can be explained more easily with constructivist developmental theories that model cognitive processes ‘from within’.^40,41^ This meta-subjective (‘from within’) perspective facilitates interpretation of psychological processes neuroscientifically, in terms of subcortical centers, cortical circuits or pathways (that express schemes) and coordinating hubs that express higher-level-complex coordinating schemes heterarchically organised—multimodal and polymodal areas and so on. Applied developmental neuroscience needs general organismic-developmental theories to organise the abundant data and reach principled semantic interpretations. Some theories exist in the literature of experimental psychology that might be seen as candidates for this organising function. Lack of space prevents us to mention them all. We take as examples dual-process theories of reasoning and cognition,^42–44^ of which fuzzy-trace theory^45,46^ is an interesting variant.

It is well known both in cognitive development and neuroscience that cognitive processes vary from (simple and fast) sensorimotor and signal processing to progressively more complex and slow, mental and symbolic modes of processing. Such gradation of progressive complexity in developmentally evolving (distinct but complementary) processes is expressed well in the cognitive-developmental stages of Piaget or Case^26,27^ among others. In neuroscience a similar gradient of variation in complementary processes is expressed on the heterarchy of functionally nested cortical areas that classic neurologists^47,48^ called concrete Primary (local sensory or motor receptor/effectors), Secondary (regional coordination sites), Tertiary (multimodal coordination hubs) and Quaternary or high Tertiary (polymodal general-control hubs). These association areas follow a sequence of progressively more abstract modes of processing that current neuroscience has renamed and much expanded regionally; but which nonetheless remain categorially (i.e., as basic functional categories, maturationally and neurologically distinct and complementary). In cognitive psychology, some related dimensions of variation have been formulated to explain why functions such as reasoning, are so variable (from concrete to abstract, from simple to complex, from intuitive/experiential to formal/verbal), from moment to moment and across subjects. Although these dimensional theories are (properly speaking) not developmental, they recognise (perhaps influenced by Piaget’s work on stages) some developmental and evolutionary aspects: simple and more concrete modes of processing occur ontogenetically and phylogenetically earlier. Influenced perhaps by Freud’s early dual-process theory—primary/unconscious processes versus secondary/conscious process—current dual-process cognitive theories have distinguished between type 1 (or system 1) mode of processing, which is fast, impulsive/automatic and global, versus the type 2 (system 2) mode of processing that is slow, reflective and analytical (monitored and controlled by working memory). A clear and balanced exposure of dual-process theories of cognition is that of Evans. As Evans^42^ states, these theories are not developmental and can only speculative talk of development (as Freud did). Further, although descriptively valid when formulating two complementary types of cognitive processing, they have problems when used in neuroscience: They are neither comprehensive enough nor describe the processes in question ‘from within’ the person’s own psychological processing. Furthermore, these theories lack developmental data, and their models are not organismic in a comprehensive way; they just discuss two important and complex descriptive modes/dimensions of variation, although there are many more (at least five others). The five categorical modes of processing we have in mind here are: (1) the intuitive meaning-bearing experiential (spatial, temporal and organismically causal) mode of processing, which may involve working memory and complex executives—related to type-2 processing and to the ‘gist’ way of fuzzy-trace theory; (2) the affective-motivational schemes and processes—not explicit in dual-type theories. (3) The explicit logical and linguistic mode of processing, which, if simple and perhaps automatised, is the Type-1 mode or the ‘verbatim’ way of processing of fuzzy-trace theory. (4) The sociocultural and sociomotivational processes, not explicit in dual-type theories. (5) Brain’s ‘hidden’ operators (polymodal controls) and executive control processes—ignored by dual-process theories. Most neuroscientists know well that each of these five modes is in fact explicitly expressed in the brain. These categorial modes of processing should be distinguished to permit comprehensive neuroscientific interpretations.

The fuzzy-trace theory^45,46^ is an interesting variant of a dual-process theory. It is interesting because its chosen dimension of variation (which has two types, gist processing versus verbatim processing) is not based on complexity, abstraction or mental effort (working memory demand), but it is instead based on meaning (deep thinking) versus the manifest signs or referents that might express simpler meaning (shallow thinking). Brainerd and Reyna refer to this contrast as the gist versus the verbatim modes of processing. This is an important dimension, both developmentally and for neuroscience. The attainment of meaning related to praxis (goal-directed activity addressed to the environment), is both the original motive and the constructivist learning guide of development. For instance, constructive-developmental schemes (the essential unit of processing that dual theories still lack) are meaning carriers, they are, from a psycho-Logical perspective^49^ context-sensitive semantic-pragmatic conditionals that are self-propelling. Also, the brain, the working brain, is a complex semantic–pragmatic processor, a meaning generator and carrier. Brainerd and Reyna^45^ also make clear that essential, effective-complexity meaning is what is abstracted from contextualised semantic-pragmatic processes: what is not essential is dropped, only the gist is retained. As we mentioned and discuss later on, the schemes activated and compatible within a situation overdetermine intended performance or representation. This overdetermination often makes representations/performances schematised and perhaps fuzzy (‘a gist’), as fuzzy-trace theory claims; this is consistent with the findings of constructivists, for whom these ‘traces’ are in fact not sensorial-perceptual traces but schemes. Representations or performances tend to become finer and clearer as more refined compatible schemes come to overdetermine the brain’s final-common-path outcomes. Unfortunately this theory does not have information-bearing units suitable for ‘from within’ processing (schemes or schemas), nor has general-purpose organismic processes (e.g., hidden operators—brain general regulations and polymodal hubs) to causally explain the emergence of gist and verbatim processes.

In the following sections, we present some challenges from developmental neuroscience data, and show some gains in adopting a constructive-developmental theoretical approach.

## To theorise developmentally or not to theorise

A challenge of developmental data is the multitude of underlying factors at play. Although, neuroscience provides rich and detailed information on core developmental processes, we are far from having clear conclusions on how the living human brain develops (maturationally and psychologically, in their interrelation). Neuroscientists often adopt non-theoretical data-driven perspectives, or else frame their work using adult neurocognitive models. Because granting agencies may value preliminary empirical findings more than theory building, rigorous theory-building might appear secondary to many neuroscientists, who may then use only adult neurocognitive models or follow data-driven approaches.

Adult findings can be used as benchmarks for how brain indices should look when mature. But, to appraise the evolving role and relative functional importance of physiological structures and processes, one needs to investigate the relevant developmental, maturational and comparative data. For instance, two related critical issues in cognitive development and neuroscience are how to formulate with clarity and explain developmental stages, and explain transitions from one stage to the next. If these issues are ignored, the child’s long metamorphosis into adulthood, and even adulthood itself, will remain unclear. Adult models cannot explain transitions and variability in human development.

**Figure 1:**
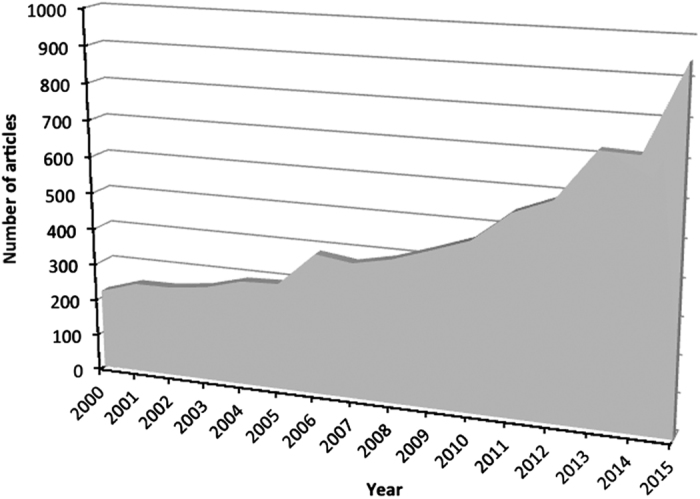
Fifteen years of publications mentioning ‘MRI’ and ‘children’ in the abstract (source: Pubmed).

It might be argued that non-theoretical, data-driven research with children avoids misinterpretation of results, which readers could then interpret. Such an approach has led to accumulation of data that remain largely unorganised. Much research is conducted in such manner. A literature search in pubmed (http://www.ncbi.nlm.nih.gov/pubmed) for the terms ‘MRI’ and ‘children’ in the publication abstract yields 8,606 papers. Figure 1 shows a breakdown by year of the number of abstracts that mention these terms in the last 15 years. There has been an exponential increase of ‘developmental’ cognitive neuroscience publications in this period.

An analogy may highlight the importance of constructivist theorising to make easier the translation of empirical science into applications. Suppose you enter an office and see it very messy. There is nothing wrong with having a messy research desk for as long as only a few persons are using it. Messy desks often are favoured by creative people. Suppose, however, that an educator or an applied developmentalist arrives looking for a red pen (which in our story might symbolise a better math curriculum or better understanding of stage-bound limits in school children’s mental attention—something important for teachers to understand). They have been told the red pen is in this office. Were the desk organised and rationally structured, the visitor could easily find the red pen. But, a messy desk has no prescribed spot for pens. Our educator or developmentalist would have to spend much time searching, and they might not find the red pen. The point of this story is that knowledge that remains primarily data-driven can create obstacles for knowledge translation. A solid, well-researched correspondence between cognitive development and neuroscience is essential, because knowledge of brain indices alone does not suffice to produce credible task analyses that would facilitate applications. Constructivist developmental theories are needed for this purpose.

## Constructivist development: What is the cause of transitions to new stages?

Theory ultimately is founded on empirical method, although initially it is a function of the theoretical method adopted, including epistemology or metatheory. When the empirical method is changed, the finding of novel repeatable functional invariances (unexpected recurrent patterns in the data) suggests that some other, perhaps complementary, explanatory account is necessary. This is the case of constructivist developmental methods when contrasted with the common experimental method dominant in adult research. The theoretical concept of developmental stages, as defined above (i.e., age-bound functional invariances in the data, coupled with a suitable explanatory organismic model) is one instance of constructive regularities, i.e., data patterns and theoretical constructs that cannot be obtained when using only experimental methods of adult research. What Piaget (and complexity theoreticians like Gell-Mann^50^) called regularities, are relevant probabilistic (often functional) invariants that emerge in the context of repeatable situations^51^ meaningful when perceived by adaptive systems like humans. Incidental random aspects may vary, but the invariant meaningful aspects are preserved over repetitions.

Some experimental neuroscience research has used mathematical methods to recognise invariant stages.^52^ Specifically, researchers have used hidden semi-Markov models for a multi-voxel pattern analysis of mathematical problem-solving tasks, which yields a sequence of invariants indexing stages of problem solving; these mathematically obtained brain processing stages confirm the rationally expected steps or stages of problem solving.^52^ Whether mathematical methods could be used to detect developmental stages in the brain is unknown, but task-analytical constructivist methods seem to detect developmental stages.^34^

For constructivists, including Piaget, effective complexity of a scheme, process or action, is given by the number of essential invariants (probabilistic, often functional, relevant regularities that emerge in the context of repeatable situations^50,51^)—invariants that are contextualised, i.e., relative to a situation and to an activity. As ordinary children grow, they can process more information, because they notice and build more complex regularities into schemes (due to growth of mental attention). Thus, the effective complexity they can cope with increases, and so they can do complex tasks more readily.^53–55^ The relationship between child development and the growth of effective complexity a person can handle was first anticipated by Binet^56,57^ who, in developing the first test of intelligence, tried to capture the child’s judgment—the ability to recognise and coordinate all task-essential aspects to synthesise the correct conclusion. He thought that with age (maturation) children can cope with more essential aspects—more effective—i.e., necessary—complexity (although this concept was only intuitive in him, and not explicated). He lacked the knowledge of how to assess effective complexity directly, and proceeded methodologically to assess complexity of item tasks by classifying items in terms of the lowest age-group that on average was able to solve the item-task in question. This passing age became for him, and also for Piaget, an empirical criterion of task complexity. This was, and remains, a safe criterion for estimating relative effective complexity of tasks, because task-analytical methods for estimating complexity more theoretically are still undeveloped, although neo-Piagetians have made progress on the matter.

However, when Terman^58^ created a psychometrically more sophisticated version of Binet’s test, the Stanford–Binet Test, he chose to dispense with this developmental method of estimating item difficulty by the passing age of children, and instead proceeded to use the probability of an item being passed by the total population, not by a particular age group. With this psychometrically practical procedure, he estimated difficulty/complexity of the items being used. Similar psychometric methods have been used for assessing item difficulty ever since, both in adults and in children. The problem with this alternative method stems from failure to distinguish between two distinct types of task that affect difficulty differently, misleading and facilitating tasks. Misleading tasks include dual-tasks paradigms^54,59^ and backward span tasks.^60,61^ Tasks are misleading (conflicting, with interfering processes) when they activate information-bearing processes such as schemes or schemas (i.e., complex representational schemes) that are mutually incompatible and compete with each other for application to the task. For instance, in a backwards digit span task the automatised habit of saying digits forward competes with the prescribed instruction of saying digits backwards. These misleading/conflicting situations involve perceptual, intellective or intellectual problem solving, and they exhibit much more clearly developmental performance-level discontinuities that empirically subtend stages of development; these are the sorts of situations that both Binet and Piaget sought. Such situations cannot be solved unless the child can attentionally inhibit misleading or irrelevant schemes, and concurrently attend mentally (with effort) to the relevant schemes needed for the intended performance, and activate them. This number of effortfully activated task-relevant schemes (not otherwise activated) is a clear index of effective complexity—in terms of an act of mental attention needed for the task. In contrast, facilitating tasks, such as standard/forward span tasks,^60,61^ can be often solved by using habitual/automatised schemes, which might not require effortful attention, or require much less.

Misleading situations, used by Binet, Piaget and neo-Piagetians, can assess effective complexity reliably—whereas facilitating situations, common in learning/memory paradigms and psychometric-intelligence tasks, do not.^62^ This is one key difference between developmental-constructivist research designs and common adult, experimental or psychometric designs. Although methods of task analysis are often lacking or omitted, appraisal of effective complexity of tasks is particularly important for developmental studies, because children performance varies with effective complexity in terms of age and individual differences. The only empirically clear way of appraising effective complexity of items or tasks is using the constructivist-developmental methods of Binet, Piaget and other constructivist researchers.^9,26^ Ignoring this methodological prescription, as happens in Intelligence Quotient measures or in common measures of working memory, leads to errors in assessing effective complexity of tasks—which may be why good theory-guided quantitative measures of effective complexity are hard to find.

Piaget’s ideas (he did follow Binet’s developmental method) were important to these investigations for decades; core assumptions to his theory have been disputed, tried and often confirmed. Piaget’s work has inspired a new generation of innovative developmental theoreticians^63^ such as Robbie Case,^36,64^ Andreas Demetriou,^65^ Graeme S. Halford^66^ and Juan Pascual-Leone,^2,13,26^ to mention only four neo-Piagetians. These and other scientists have invested decades studying developmental constructs, mechanisms and trajectories. They connected findings and organised a great deal of empirical data by using theory-guided, testable, constructivist models, in an effort to explain transitions within cognitive developmental growth.

## Two sorts of constructivist developmental (neo-Piagetian) theories

Although each neo-Piagetian approach has its unique perspective, they all explain cognitive growth as involving a progressive incrementation of effective complexity in the schemes/schemas generating performance, an incrementation concurrent with growth in processing efficiency and in working memory. Developmental constructivists are divided with respect to what causes transitions from one stage to the next. Some neo-Piagetian theoreticians and most adult working memory researchers support what we might call the Constructivist Learning group of theories. They think of developmental growth as caused by some form of insightful Constructivist Learning. Piaget would have equated constructivist learning with ‘psychogenetic/developmental intelligence,’ a construct that, for him, had four main causal factors: maturation; specific learning; general/social learning; and equilibration. Demetriou and colleagues^67,68^ call ‘Cognizance’ a related encompassing construct that, unlike Piaget’s own, seems to give primary causal power to consciousness. The analysis of causal organismic factors has been a concern of developmental constructivists, Demetriou among them. Constructivist learning often is seen as producing internalisation of recurrent functional patterns of behaviour/processing, done by way of forming schemas (i.e., complex schemes)—structures or chunks (i.e., schemes of schemes of schemes)—at various complexity levels and different content domains. Complex schemes are abstracted and coordinated, maximising efficiency both of processing and breadth of representations (indexed, with adequate experience, by the growth in working memory—Piaget’s ‘field of (mental-attentional) centration’). These theoreticians concur with Piaget in asserting that the main cause of development is fundamentally a (more or less conscious) constructivist learning; and they see working memory and speed (efficiency) of cognitive processing as secondary indexes of such constructivist growth—as Piaget would have done.^69,70^

Other neo-Piagetians—this is the Maturational Attention group of theories, agree with the importance of constructivist-learning processes but think that this sort of learning (in contrast to associative learning) is only possible by maturational growth of a limited resource: mental/executive attention.^11,13,26,36,71–73^ Maturational attention is a key determinant of working memory. For this latter group of theories, mental-attentional mechanisms grow in power as a function of age in normal children, which (along with other causes) co-determine emergence of developmental stages.

Demetriou’s current theory^65,67^ is probably the best behaviourally investigated version of the Constructivist Learning group of theories (which includes Piaget’s own). This theory claims that cognitive development occurs through domain-specific changes over at least seven specialised capacity systems: categorical thought; quantitative thought; causal thought; spatial thought; propositional thought; social-interpersonal thought; and drawing-pictographic system. These seven domain-specific systems undergo several developmental stages as these local systems constructively generate complex schemes, via ‘abstraction’ and ‘alignment’ (concepts related to Piaget’s reflective abstraction and coordination). In Demetriou’s theory, the coordination of all these complex schemes is done by encompassing executive processes and representations, which constitute a dynamic and often unconscious or preconscious functional totality that he calls ‘Cognizance’^67,68^ a concept equivalent to Piaget’s developmental (‘psychogenetic’) intelligence. Demetriou considers abstraction, alignment and cognizance the core capacities of human constructivist development. This theory could be applied in particular to compare, predict and explicate neural findings that assess brain indices (e.g., MEG and EEG) that relate to abilities such as speed of processing, within each of the seven specialised capacity systems—an individual-difference comparison that might have important educational applications. Temporally sensitive neuroscience methodologies could provide valuable data to inform and refine this developmental theory.

A theory that in its later formulation clearly belongs to the Maturational Attention group is Robbie Case’s.^36^ For him cognitive abilities improve within sub-stages by qualitatively gaining in proficiency (related to effective-complexity control), thanks to the central conceptual structures formed, via coordination and abstraction, by constructivist learning (which Case interprets using the learning constructs of Pascual-Leone^36,74^). These central conceptual structures are organised schemas (complex schemes or structures) expressing meaningful representations, relations, and acts, which underlie cognitive processes applicable across situations and across substages.^10,36,75^ Schema/scheme formation is helped by mental/executive attention; Case considered working memory to be a product of Pascual-Leone’s mental/executive attention.^2,14,36^ This working memory is the foundation allowing children to tackle progressively more complex situations, permitting transitions from one complexity level to another along four main developmental stages—each divided in four substages.^10^ Central conceptual structures may also be domain general, although Case and his associates have mostly studied domain-specific structures in the numerical, spatial and narrative domains.^10,75^ This theory would be advantageous to use in neuroscience when the research aim is to descriptively understand constructive emergence of chunks, or the progressive reorganisation of developmental schemas during infancy, childhood and adulthood. Case’s developmental methods and ideas could benefit from knowledge gained from various neuroimaging methodologies like EEG, fMRI, and/or psychophysiological methods, like eye tracking.

Pascual-Leone is the founder of neo-Piagetians, and initiator of the maturational attention group of theories.^14^ He sees Case’s important contribution as complementary to his own—as Case^36^ himself did. Case explicates some central structural aspects of Piaget’s theory.^27,74^ Pascual-Leone’s Theory of Constructive Operators (TCO) was developed as a causal model of organismic processing that can explain Piaget’s descriptive theory of development—a theory that other neo-Piagetian theories helped to clarify. Instead of adopting, as most theories do, an observer’s perspective, the TCO adopts the perspective of the subject’s own processes: a perspective from within the subject-agent that he calls metasubjective.^41^ A key contribution of this approach, relative to others, is to offer a theory-guided method of fundamental measurement for mental/executive attention. This method uses process-task analysis to quantify the mental-attention demand of tasks, and also the mental attentional capacity available to average children in each qualitative stage of development. Such quantification of stages is important, because it allows parametric evaluation, on a graded scale, of effective complexity in mentally demanding tasks, such as problem solving in misleading situations.^2,13,14,26^ Within this theory, behaviour is generated by the mediation of various general-purpose ‘hidden’ operators or brain resources (such as mental attentional effort, the field factor of simplicity—the brain’s lateral inhibition—the learning mechanisms and so on), which modulate functioning and activation of self-propelling schemes. All this governed by a principle of overdetermination that the activated schemes—information-bearing circuits—follow.^2,14,21,41,76^ This principle is related to Tolman’s^77,78^ and Freud’s dynamic conceptions of motivation and behaviour, and can be explained by the spreading of neuronal activation among connected neurons, and by an organismic generalisation of Sherrington’s neural principle of a final common path,^79,80^ coupled with Edelman’s model of brain’s lateral inhibition.^81^ This principle of overdetermination states that at any moment performance is determined by the dominant set of activated compatible processes (schemes), often in competition with other activated interfering processes. Such theory helps to clarify the trade-off between cognitive load and mental-attentional capacity: children cannot solve a task if its cognitive load (number of relevant schemes that need activation) is above the child’s mental-attentional capacity.^2,14^ The highest maturational mental-capacity of a child is the pivot point of this trade-off; it indicates, at least within misleading situations, a developmental stage level that the child has reached. Mental-attentional capacity increases every other year after the age of three, reaching a mental-attentional capacity of seven units at 15–16 years (this count of 7 is obtained only when all necessary schemes, figurative as well as operative and their essential parameters, have been counted). This maximal capacity of 7 is equivalent to that of adults,^82^ although adults often function mobilising at most a capacity of 5—unless highly motivated and challenged. This theory would be advantageous in its neuroscience application in at least four main categories: (1) It can help to understand better the person’s cognitive-complexity constraints or limitations (within and across domains), because mental-attention is a general resource measurable across domains (at best, attaining interval scales), which yields related units of measurement comparable across domains. (2) It can help to appraise and explicate the effective complexity of different levels of processing abstraction (from experiential to high conceptual, irrespective of the domain of application). These levels are clearly visible in the brain’s organisation (e.g., from primary modalities to regional modal hubs, to multimodal hubs, to polymodal integration hubs—plausible sites of consciousness) and thus the TCO can help to put in a more rigorous footing these orders of semantic complexity in the brain processing. (3) It can help to *explain* neural findings anchored on age-specific cognitive limits or individual-difference styles. (4) It can help to conceptualise and interpret brain processes with reference to complex relations between cognitions (truth-evaluation schemes—lateral frontal, parietal, temporal and full occipital, lobes), and affects or emotions (organismic-feeling-evaluation schemes—ventral and medial frontal, parietal, temporal lobes), versus motivation (the conversion of affective propensities or goals into cognitive goal or plans—which involve areas such as insula, amygdala, orbitofrontal cortex, anterior cingulate gyrus.^29^ Some work in this direction has provided important neuroimaging evidence regarding the brain-behaviour relations in terms of cognitive-complexity constrains. However, further neuroscience research using constructivist-developmental models could directly evaluate core hypotheses of constructivist theory (e.g., domain-general versus domain-specific processes, individual differences styles and boundaries between cognition and affect).

Halford *et al.*^66,71,83^ have also focused on how to assess the effort/load of mental/executive attention. They attempt this appraisal by estimating effective complexity in terms of the number of inter-related terms that must be jointly considered in the task at hand—relational complexity. This Relational Complexity Theory states that meaning occurs when a link is formed via inter-relations (e.g., ‘cat’ and ‘lion’ are related because both are felines, and one is smaller than the other, i.e., two binary relations; and might also be related because ‘their particular trainer prevents the lion from attacking the cat'—a ternary relation). Meaning accrues when higher order relations (unary, binary, ternary, quaternary relations and so on) get formed. However, Halford’s relational complexity is not the only task-relevant sort of effective complexity; tasks’ mental demand can also accrue with other not inter-related but relevant relations or pieces of knowledge, such as those that must be kept in mind for later use in a task. Nonetheless relational complexity is perhaps the most important aspect of effective knowledge, as Cassirer^84,85^ early emphasised. Indeed, relational complexity expresses the rank (semantic complexity) of relations involved in tasks, which increases task difficulty in children, relative to age. Halford’s theory and method would be particularly useful to researchers who seek neural findings related to language, and linguistic complexification. It could also be very useful in research about how/why (or under which conditions) information processing generates task complexity—using neuroscience as source of dependent variables while Halford’s relational complexity is being varied. Also, to investigate how/when the person’s repeated practice on relational-complexity tasks (each varying in semantic ranking) can lead to the behavioural emergence of chunks, and to concurrent change in the dominant cortical sites of processing.

When these and other constructivist developmental theories are compared.^63^ it is apparent that they share many conceptual assumptions over when, how and what constructs develop as a function of age—assumptions with considerable empirical support. The backbone of these theories is a mental-attentional growth that in adulthood is completed, although the brain still develops and learns. These empirical landmarks are critical moments, i.e., transitions in the coping capacity for effective complexity.

Some of these theoreticians attempted to articulate their constructs in terms of brain regions and processes.^10,14,21,22,67,71^ Clarity, however, is missing about how and when relations between brain and behaviour begin to appear, preparing children for one or another school-grade activity. Real significant applications in Education and learning environments are more likely to emerge when neuroscience, constructivist-developmental work and cognitive psychology are brought together to clarify issues.^86–88^ An important determinant for building these links is the methodological choices made in research.

## Developmental cognitive neuroscience: some concrete methodological considerations

Constructivist-developmental theory is needed to guide *a priori* methodological choices. For instance, we know the major cognitive changes between early childhood and adolescence from theories of cognitive development. Nonetheless, many neuroimaging studies practice averages over excessively large age ranges, thereby cancelling possibilities of detecting variations across groups of children, obtaining instead spurious averages. Consistent patterns of variability over developmental groups exist already, with empirical indicators for general cognitive-developmental constructs, which help to explain ontogenetic emergence (adults are grown up children!). For instance, when in an fMRI study children from 7 to 13 years are tested, should their brain responses be averaged with those of 10-year olds? A constructivist developmental theory would advise against such averaging, because age-specific changes in core cognitive processes do occur in that interval, which could empirically help to clarify some theoretical constructs. Consider one inference stemming from Pascual-Leone’s theory. The Theory of Constructive Operators predicts (with much cross-sectional and some longitudinal support) that one symbolic-processing unit (i.e., units suitable for the symbolic processing that do not exist in infancy, which only has sensorimotor scheme units) of change in mental-attentional capacity (Mc) occurs every other year after the age of 3 years; reaching a total of seven (symbolic-processing) units that can be simultaneously processed at about 15–16 years. However, within misleading situations, performance level is function of the trade-off between task’s mental demand (Md) and the Mc of participants: performance should increase or decrease with this difference (Mc minus Md); and participants should fail (other things equal) when Md is higher than Mc.^2,14,26^ Thus, particularly in misleading situations, and depending on the desired topic of study, the age-range and age-group selections should be varied concurrently with the task’s Md; and only if the difference Md minus Mc is kept constant across age-samples the performance (or practical) complexity of tasks will have been kept invariant across age groups.^9^ In sum, choice of age groups in multiple-age experiments, and the expected performance complexity within or across age groups, should be guided by developmental theory.

There are other aspects of task selection that are methodologically critical. In functional imaging studies, tasks must adhere to specified limitations posed by physical properties of the imaging method used.^86–89^ For instance, tasks in fMRI need to minimise any form of physical-body movements, because this would negatively affect image quality. At the same time tasks should assess the desired construct without reaching ceiling or floor effects (or producing chance performance) for every age group. These controlled constraints induce variability of performance over participants, increasing the power of intended relevant tasks. No child would easily remain in an MRI scanner to complete a long, monotonous task. Tasks designed for children must be short (e.g., between 30 to 60 min, depending on the age of the child), have shorter runs (e.g., four 5-min runs/experiments instead of two 10-minute runs/experiments) friendly and engaging, but with enough trials (e.g., 32 trials per category) to obtain measurable developmental effects. And to ensure that the task is age appropriate, and verify that it elicits the theory-expected age-differential performance, tasks for children should be task-analysed and validated behaviourally outside the scanner.^9,88^

Age-variable performances call for parametric task designs. This need has been identified,^90^ but it is rare to see parametric protocols in developmental neuroscience.^91,92^ In constructivist cognitive development, behavioural quantitative measures of mental attention, with scaled levels of effective complexity, have been used for years. They assess cognitive limitations as a function of age.^2,12–14,26,93^ Well-constructed measures, which quantify performance-differences parametrically, are valuable tools for neuroimaging.^88^ We elaborate on two main reasons. First, parametric studies using an interval-scale metric allow construction of difficulty levels that accommodate age-dependent differentiation of performance—thus quantifying developmentally cognitive stages.^2,9,13,14,26^ It is important that difficulty levels be consistent and gradual; we are not thinking of tasks such as the usual n-back task where difficulty increases across three qualitative levels with irregular difficulty grading. Tasks with gradual increases of effective complexity have invariant executive requirements across suitably-graded levels (to separate tasks’ executive demand from maturational attention demand); and so increments in cognitive load can be graded in mental-attention demand, varying with the number of items being processed.^9,14,26^ This constructivist method ensures that both younger and older children can perform the task well: executive requirements are the same and task items exhibit suitably graded differences, which adjust the performance’s relative complexity (the Mc-versus-Md trade-off) in a natural way. We say a natural way because complexity levels in such cases are not arbitrary; they can be empirically determined by performance passing levels of age-group samples, in suitably chosen misleading tasks. Second, parametric scaling and this natural metric (constructivist-developmental—heir of Binet’s old method) offer new options for analysing brain responses. For example, within-group differences can be examined in a scaled manner as a function of item difficulty.^34^ Brain responses could be analysed based on both observed performance level of the individual child and the group’s scaled average across age groups. Tasks with parametric scaling also allow for examination of brain responses vis-a-vis item difficulty—as a function of age. Alternatively, brain responses can be analysed based purely on the constructivist-developmental theoretical expectations.

Functional neuroimaging offers powerful methods to measure brain responses vis-a-vis cognitive tasks within and across stages; but methodological choice rests on the hands of researchers, who want to maximise their intended yield of the interpreted data. To this end, it is not only critical to adopt a developmental methodology, but important to have constructivist-developmental theories.

## Conclusion

Children think and act with the brain processes they have; age-bound changes in typical behaviour are pointers to neuroscientific theoretical constructs we should adopt. However, funding agencies often finance projects involving children with atypical development, with much less funds invested in typical development. There are nonetheless magnificent exceptions such as the IMAGEN study,^94^ the Pediatric Imaging, Neurocognition, Genetics Study (PING),^95^ the study on the Philadelphia Neurodevelopmental Cohort (PNC),^96^ and a recent National Institute on Drug Abuse (NIDA)-funded project on Adolescent Brain Cognitive Development (ABCD). These are efforts in developmental neuroscience producing fascinating findings. But, the lack of systematic framing and synthesis within a constructivist developmental theory could turn into an Achilles’ heel for developmental cognitive neuroscience. Developmental methodology in children’s neuroscience has been encouraged in clinical research with children with mathematical deficits^97^, dyslexia^98^ and other neurodevelopmental disorders.^99,100^ However, developmental methodology needs to be at the heart of developmental cognitive neuroscience for typical and atypical development. We propose that the field will experience positive growth by using knowledge from constructivist-developmental frameworks in this manner: (a) age groups selection will be theory-based; (b) tasks processes can be (i) modelled using a ‘from-within subjects’ perspective; and (ii) are measured parametrically; and (c) inferences are process-analytical so as to be comparable across studies. Finally, neuroscientists must think more critically about how their research is portrayed to educators, and how it may be translated in clinical practice. They should consider benefits of working towards a comprehensive developmental framework versus taking a study-by-study approach.

We believe that when developmental methods become more widely used, theory building may occur more naturally, and foster syntheses of developmental cognitive neuroscience data. Ultimately, a bridge between neuroscience and constructivist developmental theory should bring about collaborative relationships among researchers, and facilitate communication with the public.
